# Pavement Performance Investigation of Asphalt Mixtures with Plastic and Basalt Fiber Composite (PB) Modifier and Their Applications in Urban Bus Lanes Using Statics Analysis

**DOI:** 10.3390/ma16020770

**Published:** 2023-01-12

**Authors:** Xueyang Jiu, Peng Xiao, Bo Li, Yu Wang, Aihong Kang

**Affiliations:** 1 College of Civil Science and Engineering, Yangzhou University, Yangzhou 225127, China; 2 Research Center for Basalt Fiber Composite Construction Materials, Yangzhou 225127, China

**Keywords:** asphalt mixture, plastic and basalt fiber composite modifier, urban bus lane, pavement performance, statics analysis

## Abstract

A new type of plastic and basalt fiber composite (PB) modifier, which is composed of waste plastic and basalt fiber using a specific process, was used for bus lanes to address severe high-temperature deformation diseases due to the heavy loads of buses. The dense gradations of asphalt mixture with a nominal maximum aggregate size of 13.2 mm (AC-13) and 19 mm (AC-20) were selected to fabricate asphalt mixtures. The impact of the modifier PB on the high-temperature rutting resistance, low-temperature crack resistance, and water damage resistance was investigated experimentally. The experimental results showed that adding the modifier PB could enhance the rutting resistance and water damage resistance of asphalt mixtures significantly while maintaining the low-temperature crack resistance. Then, PB-modified asphalt mixtures of AC-13 and AC-20 were employed into a typical pavement structure of a bus lane in Yangzhou city, China, and three types of designed pavement structures were proposed. On this basis, statics analyses of all of the designed structures were performed using the finite element method. The statics analyses revealed that, compared with the standard axle load, the actual over-loaded axle made the pavement structure of the bus lane suffer a 30% higher stress and vertical deformation, leading to accelerated rutting damage on the bus lanes. The addition of the modifier PB could make the pavement structure stronger and compensate for the negative effect caused by the heavy axle load. These findings can be used as a reference for the pavement design of urban bus lanes.

## 1. Introduction

More than 230 cities in China have created priority lanes or bus-only lanes, according to the preferential policies and measures providing priority for the development of public transport, to alleviate urban traffic congestion [1]. However, there is a lack of guidance for the design of the bus-only lanes. The current research on urban bus lanes is mainly focused on the route design, traffic flow calculation, traffic organization optimization, etc. The bus-running characteristics are seldom taken into account for the pavement structure design [2,3,4]. It is reported that the tire ground pressure of a bus normally exceeds 0.7 MPa, which is the standard axle load for urban pavement structure design in China according to the specification of CJJ 37-90. Subsequently, pavement distresses such as rutting, upheaval, etc., appear frequently on bus-only lanes due to the insufficient structure design [5]. Therefore, new material selection and (or) design structures of bus lanes are desired.

Waste plastics are considered as an effective modifier for asphalt since not only can they strengthen the pavement performance of asphalt mixtures [6,7,8], but they can also provide a solution to the environmental pollution caused by waste plastics [9,10]. Nouali et al. [11] examined the suitability of using plastic bag waste as a bitumen modifier in order to improve the behavior of asphalt mixtures. The results show that adding plastic waste to the pure bitumen allows for reducing the void content of the mix and substantially increasing its stiffness modulus and water resistance. In Ranieri’s [12] study, the rut depth values were reduced by more than 30% for waste HDPE-modified asphalt mixtures compared with conventional asphalt mixtures. Padhan’s and Shahane’s research [13,14] indicates that plastic gives an increase in stability, compressive strength, and split tensile strength compared to the conventional SMA/AC mixtures. It was reported that waste plastics modifiers could improve the adhesion between asphalt/aggregates and enhance the high-temperature performance of asphalt pavements [15,16]. However, adding a waste plastics modifier would also make the asphalt mixture prone to cracking, leading to a deterioration in the low-temperature performance [17,18]. Therefore, the composite modification method becomes more popular when waste plastics are used as the modifier for asphalt pavements.

Basalt fiber (BF), as a new, environmentally friendly mineral fiber [19,20], has been widely used in asphalt pavement due to its unique advantages: a wide working temperature range, excellent mechanical properties, chemical stability, anti-aging and thermal insulation properties, etc., [21]. In Celauro’s and Hui’s research [22,23], basalt-fiber-modified asphalt mixtures show a better high-temperature performance with reference to permanent deformation resistance when compared with the traditional mixture. Moreover, the chopped basalt fiber is combined with the asphalt well and distributed in a three-dimensional network structure in asphalt mixtures, which can reinforce the low-temperature performance of asphalt mixture significantly [24,25,26]. Zhu et al. [27,28] investigated the anti-fatigue property of basalt-fiber-reinforced asphalt mixture. The results present that, after adding basalt fiber, the cumulative dissipation energy is greatly improved, and then the fatigue life of the asphalt mixture is increased. The impact of the dosage and fiber length on the asphalt pavement performance was also investigated by laboratory tests, and the optimum fiber dosage and applicable fiber length for the dense graded asphalt mixture of AC-13 were recommended as 0.2~0.4% and 6 mm [29,30,31,32]. In addition, relevant specifications have also been issued to guide the application of basalt fibers in asphalt pavements [33,34]. It was pointed out that the addition of basalt fiber could enhance the high-temperature stability and low-temperature property of asphalt mixtures; in particular, the fatigue performance can be remarkably prolonged. However, the improvement effect on the high-temperature property of asphalt mixtures is inferior to that of waste plastics. If the addition of waste plastics and basalt fibers can give full play to their respective advantages in different performances of asphalt mixtures, then the waste-plastic–basalt-fiber-modified asphalt mixture can be well used to address the common pavement distresses of bus lanes.

Based on the self-developed composite modifier PB for an urban bus lane, the objective of this study was to investigate the high-temperature performance, low-temperature property, moisture stability, and dynamic modulus of PB-modified asphalt mixtures. In addition, taking the bus lanes in Yangzhou city as an example, a statics analysis of the bus lane structure was conducted using the finite element method. The findings of this study can be used as a reference for the pavement design of urban bus lanes.

## 2. Raw Materials and Experimental Methods

### 2.1. Raw Materials

#### 2.1.1. Modifier PB

**Figure 1:**
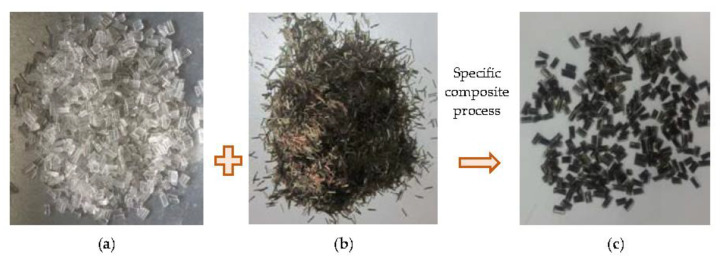
Illustration of modifier PB. (**a**) Waste plastic synthetic particles; (**b**) basalt fiber; (**c**) modifier PB.

The modifier PB was made of waste plastic and basalt fiber with a weight ratio of 1:1, and manufactured using a specific process. The main source of waste plastics is waste polythene, and the components and contents are shown in Table 1. Basalt fiber is produced by Jiangsu Tianlong Basalt Fiber Co., Ltd., Yangzhou, China, and the properties are listed in Table 2. The modifier PB has colorless transparent surface and a brown metallic luster inside as flat solid particles, as shown in Figure 1c, and can be stored at ambient or low temperature. As PB contains basalt fiber component, according to the previous finding that the optimum basalt fiber length for asphalt mixtures is 6 mm [24,26], the length of the modifier PB was determined to be 6 mm in this study. Its technical indicators are shown in Table 3.

#### 2.1.2. Aggregates and Mineral Filler

Limestone was used as the aggregates in this paper. The mineral filler selected was limestone powder, and the technical properties are shown in Table 4.

#### 2.1.3. Asphalt

Both base asphalt and styrene butadiene styrene (SBS)-modified asphalt, produced by Tongsha asphalt factory in Jiangsu province, were adopted in this study. The technical properties of the two types of asphalts are shown in Table 5.

### 2.2. Gradation Design

#### 2.2.1. Gradation Curve

**Figure 2:**
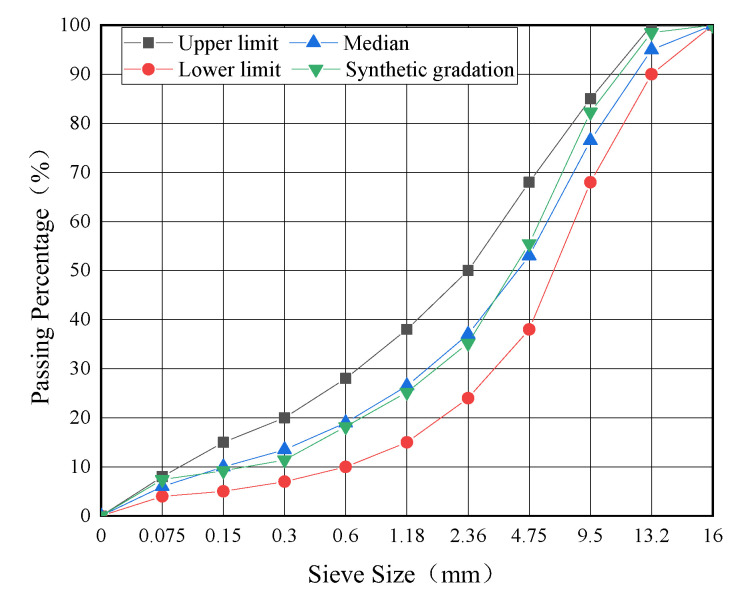
Gradation curve of AC-13.

**Figure 3:**
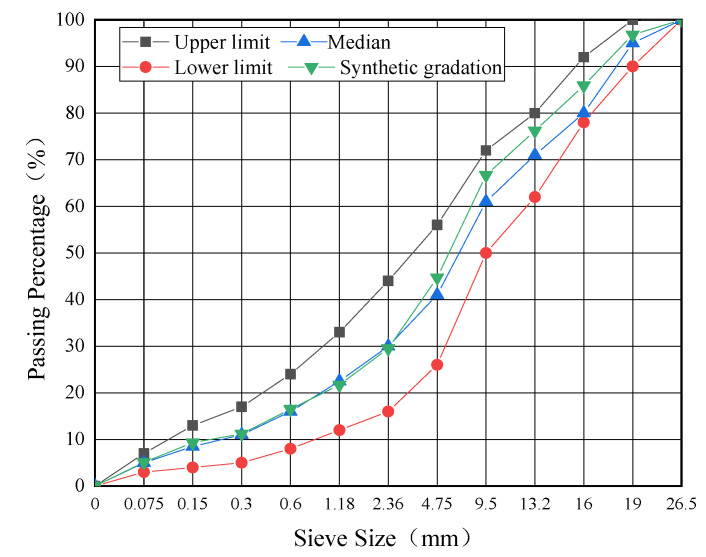
Gradation curve of AC-20.

Two types of dense gradations of AC-13 and AC-20 that were widely used in the bus lanes in Yangzhou city (Jiangsu Province, China) were selected in this study. The gradation curves of the two types of asphalt mixtures were illustrated according to JTG F40 [35], as shown in Figure 2 and Figure 3.

#### 2.2.2. Volumetric Properties of the Asphalt Mixtures

According to the difference in asphalt and modifier, three types of asphalt mixtures with a gradation of AC-13 were fabricated, called base asphalt + AC-13, base asphalt + AC-13 + PB, and SBS-modified asphalt + AC-13, respectively. The same three types of asphalt mixtures with a gradation of AC-20 were also fabricated. It is worth pointing out that the “dry mixing process” was adopted to fabricate the PB-modified asphalt mixtures, which means that the modifier PB was mixed with heated aggregates at a mixing temperature of 175 °C for 90 s before adding asphalt according to JTG E20-T0702 [36]. The dosage of the modifier PB in asphalt mixtures was 0.6% by the weight of aggregates. The related volumetric properties and the optimum asphalt content (OAC) of all six types of asphalt mixtures were determined by the Marshall design method in accordance with JTG F40 [35]. The results are shown in Table 6.

### 2.3. Test Methods

#### 2.3.1. High-Temperature Stability Test

Wheel-tracking test and dynamic creep test were used to appraise the high-temperature stability of asphalt mixtures.

The wheel-tracking test was conducted in accordance with JTG E20-T0719 [36] under a test temperature of 60 °C and test tire ground pressure of 0.7 MPa. The dynamic stability (*DS*), relative deformation ratio (*RDR*), and comprehensive stability index (*CSI*) were adopted to assess the rutting resistance of asphalt mixtures, which were calculated by Equations (1)–(3), respectively. Generally speaking, higher *DS*, smaller *RDR*, and higher *CSI* values can guarantee a better rutting resistance of asphalt mixtures.

(1) $$DS=\frac{(t_{2}-t_{1})\times N}{d_{2}-d_{1}}\times C_{1}\times C_{2}$$

(2) $$RDR=\frac{\Delta L}{D}\times 100\%$$

(3) $$CSI=\frac{(t_{2}-t_{1})\times N}{(d_{2}-d_{1})\times d_{1}}$$

where: *d_1_* and *d_2_* are the deformation of the asphalt mixture at the running time *t_1_* (45 min), *t_2_* (60 min), mm; *C_1_* and *C_2_* are test coefficients, *C_1_* = *C_2_* = 1.0; *N* is the running speed of the wheel, usually 42 times/min; *ΔL* is the depth of rutting of the asphalt mixture under load at specific timing, mm; *D* is the total thickness of the specimen, mm.

**Figure 4:**
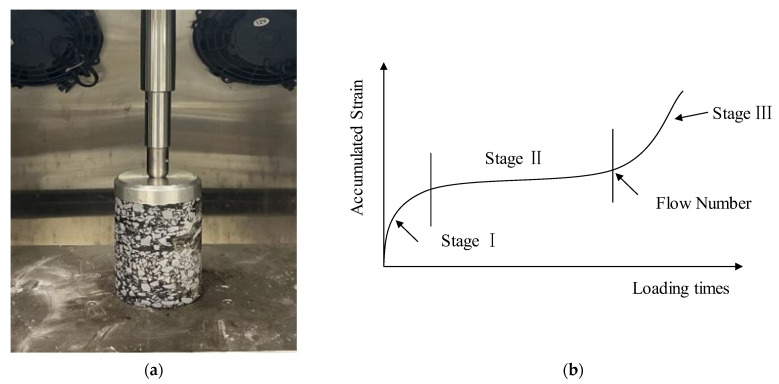
Dynamic creep test illustration. (**a**) Dynamic creep test process; (**b**) illustration of three stages.

In addition, a dynamic creep test was performed in accordance with the method described in NCHRP9-29. Three test temperatures of 40 °C, 50 °C, and 60 °C were selected. The test would end until the cumulative permanent strain reached 50,000 με or the cumulative loading time reached 10,000 s. The test process and typical strain-loading time curve are shown in Figure 4. The flow number and creep rate were served to assess the high-temperature deformation performance of asphalt mixtures. As shown in Figure 4b, the accumulated strain can be divided into three stages: creep migration (Stage Ⅰ), creep stability (Stage Ⅱ), and creep failure (Stage Ⅲ). Generally, the slope of the linear growth curve in Stage Ⅱ is regarded as the creep rate, and the flection point between Stage Ⅱ and Stage Ⅲ is regarded as the flow number.

#### 2.3.2. Low-Temperature Performance Test

**Figure 5:**
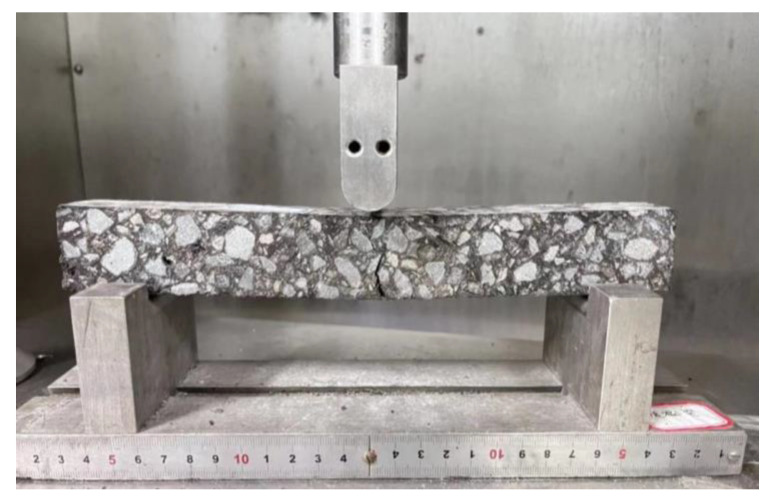
Low-temperature bending beam test.

According to the Chinese test procedure JTG E20-T0715 [36], low-temperature bending beam test was adopted to explore the low-temperature property of asphalt mixtures. The loading speed was 50 mm/min and the test temperature was −10 °C. The flexural-tensile strength (*R_B_*), failure strain (*ε_B_*), and flexural stiffness modulus (*S_B_*) were calculated according to Equations (4)–(6). Normally, the higher the flexural-tensile strain and lower flexural stiffness modulus are, the better the low-temperature cracking resistance of asphalt mixtures will be. The test process is illustrated in Figure 5.

(4) $$R_{B}=\frac{3LP_{B}}{2bh^{2}}$$

(5) $$\varepsilon_{B}=\frac{6hd}{L^{2}}$$

(6) $$S_{B}=\frac{R_{B}}{\varepsilon_{B}}$$

where: *b* is the width of the specimen, mm; *h* and *L* are the height and span diameter of the specimen, mm; *P_B_* is the maximum load on the specimen, N; *d* is the span deflection at failure point, mm.

#### 2.3.3. Water Stability Test

In accordance with the Chinese test procedure JTG E20-T0709 and T0729 [36], the water stabilities of asphalt mixtures were evaluated by immersion Marshall test and freeze–thaw splitting test. Residual stability (*MS_0_*) and freeze–thaw splitting tensile strength ratio (*TSR*) were used to estimate the water damage resistance of asphalt mixtures, which are defined by Equations (7) and (8), respectively.

(7) $$MS_{0}=\frac{MS_{1}}{MS}\times 100$$

where: *MS*_1_ is the conditioned stability of the samples that endured hot water immersion (60 °C, 48 h), kN; *MS* is the unconditioned Marshall stability of the samples, kN.

(8) $$TSR=\frac{R_{T2}}{R_{T1}}\times 100$$

where: *R*_*T*2_ is the average value of the splitting strength of the conditioned samples, MPa; *R*_*T*1_ is the average value of the splitting strength of the unconditioned samples, MPa.

#### 2.3.4. Dynamic Modulus Test

The dynamic modulus test was conducted according to the Chinese specification of JTG E20-T0738 [36]. Since dynamic modulus is temperature and frequency-related, four different test temperatures of 5 °C, 20 °C, 40 °C, and 55 °C were selected in this study. With respect to the frequency, it is reported that the frequency caused by the vehicle moving is related to its speed, pavement evenness, etc., [37]. In accordance with the data provided by the Yangzhou Passenger Transport Management Office, the average speed of the bus is approximately 30 km/h, which caused a corresponding load frequency of around 0.1 Hz [38]. Therefore, a load frequency of 0.1 Hz was adopted in this study.

For all of the above mentioned tests, the average values of three duplicate samples were used for the results analyses and discussions.

## 3. Results and Discussion

### 3.1. High-Temperature Stability

#### 3.1.1. Wheel-Tracking Test Results

**Figure 6:**
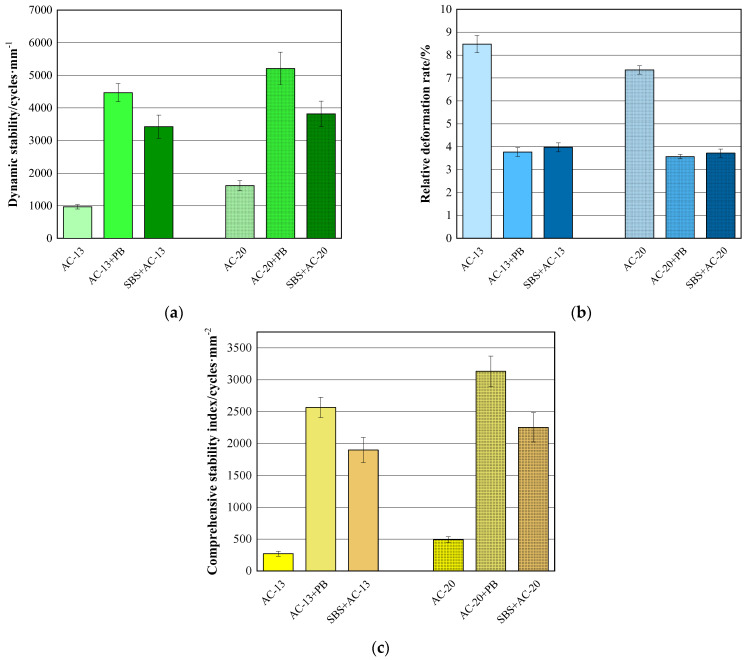
Results of wheel-tracking test. (**a**) Results of dynamic stability; (**b**) results of relative deformation rate; (**c**) results of comprehensive stability index.

The results of the *DS*, *RDR*, and *CSI* of the wheel-tracking test are shown in Figure 6. It can be drawn from Figure 6 that the addition of modifier PB can improve the *DS* and *CSI* significantly, while reducing the *RDR* to some extent. As for the asphalt mixtures with AC-13 gradation, the *DS* of the PB-modified asphalt mixtures increased by 3.6 times, while the *CSI* increased by 8.5 times and *RDR* decreased by 4.7 percentage points compared with the base asphalt mixtures. Furthermore, compared with SBS-modified asphalt mixtures, the *DS* and *CSI* of the PB-modified asphalt mixtures also increased dramatically by 30.5% and 35.1%, respectively, even though the *RDR* remained at the same level. As for AC-20-graded asphalt mixtures, the same trends of the wheel-tracking test results could be observed when the modifier PB was used. Those findings indicate that the modifier PB possesses a superior capability to improve the high-temperature stability of asphalt mixtures, even much better than the SBS-modified asphalt. One reason could be that waste plastics can increase the viscosity of the asphalt binder, leading to an improvement in the stiffness modulus of asphalt mixtures [10,11]. The other reason could be that basalt fiber can form a three-dimensional network structure, which restricts the plastic deformation of asphalt mixtures and enhances the shear resistance of the mixtures at high temperatures [24,25].

#### 3.1.2. Dynamic Creep Test Results

**Figure 7:**
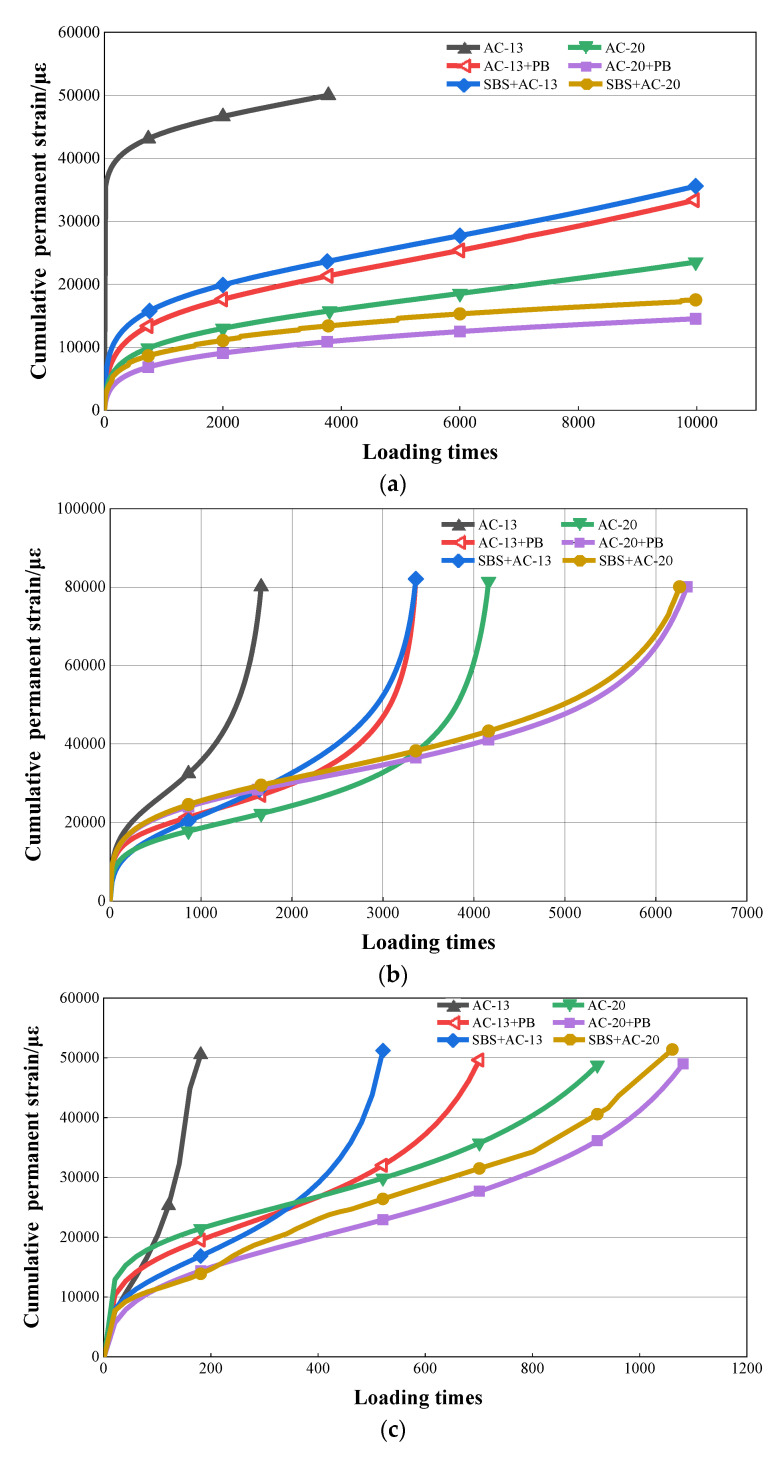

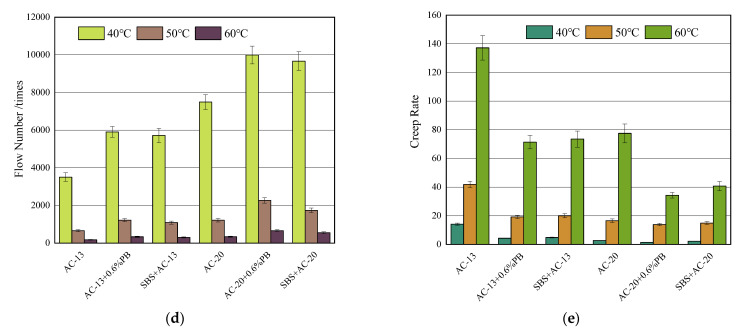
Results of dynamic creep test. (**a**) Cumulative permanent strain versus loading times at 40 °C; (**b**) cumulative permanent strain versus loading times at 50 °C; (**c**) cumulative permanent strain versus loading times at 60 °C; (**d**) flow number; (**e**) creep rate.

Figure 7 lists the results of the dynamic creep test. As shown in Figure 7, both the flow number and creep rate were temperature-dependent. The flow number values of all types of asphalt mixtures decreased with the increase in temperature, whereas the creep rate values presented an increasing trend. At the same temperature, for both AC-13 and AC-20 gradations, adding modifier PB could greatly enhance the flow number values of asphalt mixtures. The flow number values of different mixture gradations presented a similar trend in the following order: base asphalt mixture < SBS-modified asphalt mixture < base asphalt mixture with PB.

As for the asphalt mixtures with AC-13 gradation, the flow number values of PB-modified asphalt mixtures increased by 68.5%, 85.8%, and 89.8%, whereas the creep rate values decreased by 69.7%, 54.1%, and 47.9% at the three test temperatures of 40 °C, 50 °C, and 60 °C, respectively, when compared to those of base asphalt mixtures. Even compared with the SBS-modified asphalt mixtures, a positive improvement could also be observed in the flow number values and creep rate values of PB-modified asphalt mixtures. In addition, similar trends could be observed when modifier PB was used for the asphalt mixtures of AC-20. Compared with the base and SBS-modified asphalt mixtures, the flow number values of the PB-modified asphalt mixtures increased to as high as 92.1%, whereas the creep rate values reduced by 55.7% for the maximum. 

These findings infer that adding modifier PB can significantly strengthen the permanent deformation resistance of asphalt mixtures in terms of creep deformation. The higher the temperature, the greater the enhancement achieved.

### 3.2. Low-Temperature Crack Resistance

**Figure 8:**
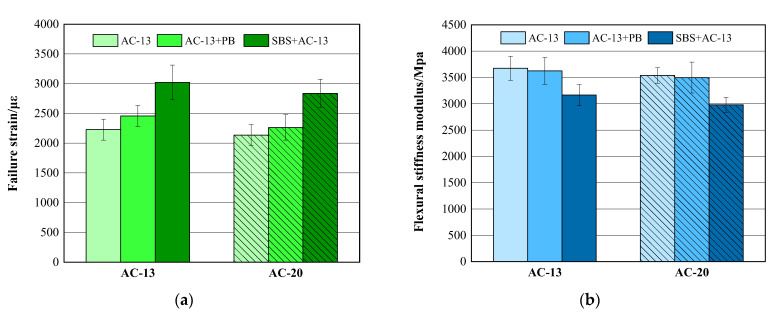
Low-temperature bending beam test results. (**a**) Failure strain; (**b**) flexural stiffness modulus.

The results are illustrated in Figure 8. It can be observed that, when modifier PB was used, the failure strain values of both AC-13 and AC-20 asphalt mixtures increased by 10.3% and 6.0%, respectively, compared with the corresponding base asphalt mixtures. Meanwhile, the flexural stiffness moduli presented a slightly decreasing trend. It can also be seen that the failure strain values of SBS-modified asphalt mixtures for both AC-13 and AC-20 gradations were much higher than those of PB-modified or base asphalt mixtures. These findings indicate that modifier PB does enhance the low-temperature crack resistance of the mixtures to some extent. As mentioned before, the addition of the waste plastics modifier would only make the asphalt mixtures sensitive to cracking, despite the positive impact on the high-temperature performance. By combining waste plastics with basalt fiber, the new type of modifier PB eliminates that negative effect on the low-temperature anti-cracking performance. In addition, the failure strain of the PB-modified asphalt mixture exceeded 2000 µε, which met the requirements of JTG F40 [35].

### 3.3. Water Damage Stability

**Figure 9:**
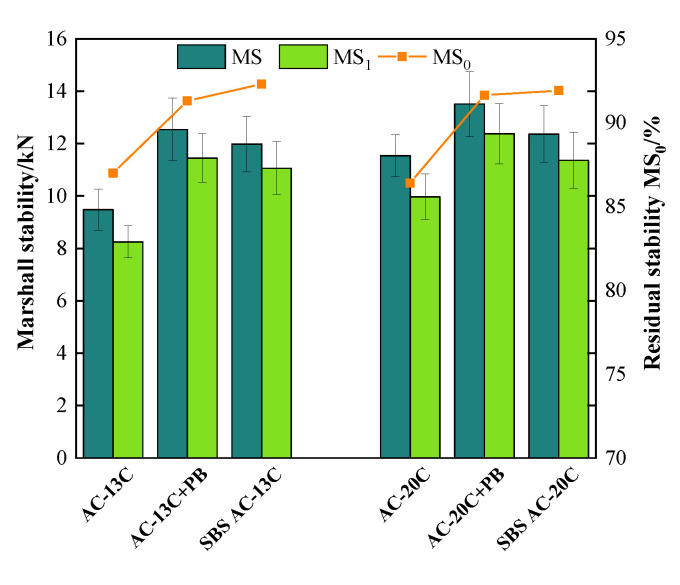
Results of water immersion Marshall test.

**Figure 10:**
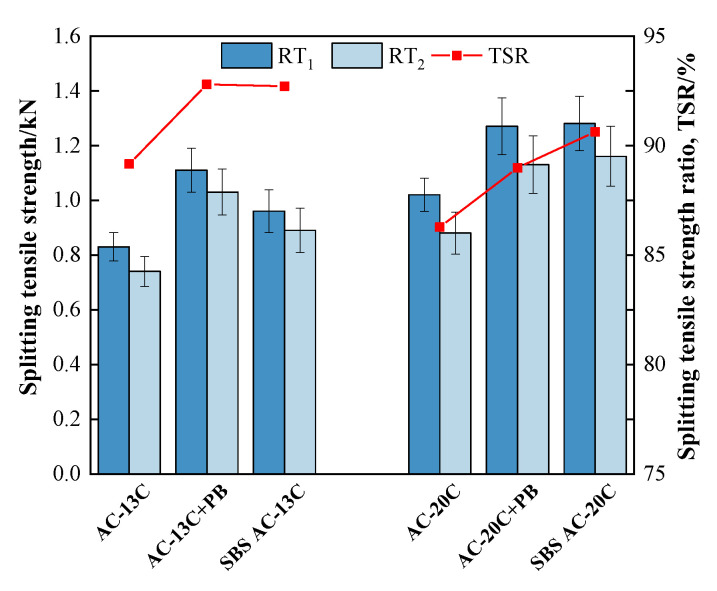
Results of freeze–thaw splitting test.

Figure 9 and Figure 10 illustrate the results of the water stability tests. It can be seen that the *MS_0_* and *TSR* of all types of asphalt mixtures exceeded 85%, which met the requirements in the Chinese specification of JTG F40. Furthermore, adding modifier PB not only improves the *MS_0_* values or *TSR* values of mixtures but also strengthens the absolute values of the Marshall stability or splitting tensile strength. 

In terms of the mixtures of AC-13 gradation, compared with base asphalt mixtures, the *MS_0_* values of PB-modified mixtures increased from 87.0% to 91.3% while the *TSR* increased from 89.2% to 92.8%, which were comparable to those of SBS-modified mixtures. In addition, the unconditioned Marshall stability (*MS*) of PB-modified mixtures grew from 9.48 kN to 12.54 kN, while the unconditioned splitting tensile strength *R_T1_* rose from 0.83 MPa to 1.11 MPa. In terms of the mixtures of AC-20 gradation, similar trends could be observed when modifier PB was used. These findings mean that adding modifier PB can significantly boost the water damage stability of asphalt mixtures.

### 3.4. Dynamic Modulus

**Figure 11:**
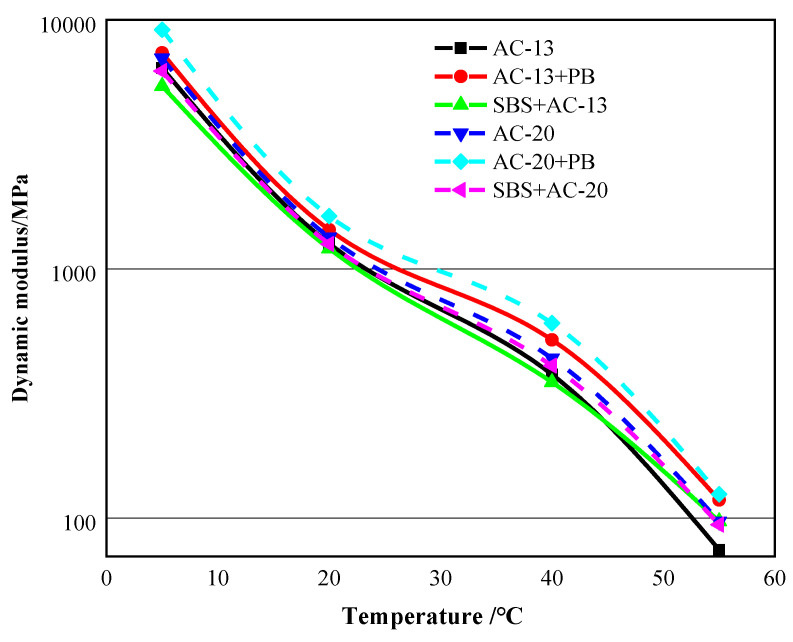
Results of dynamic modulus test (test frequency: 0.1 Hz).

The results of the dynamic modulus test are shown in Figure 11. It was noticeable that the dynamic moduli of the asphalt mixtures with modifier PB maintained the highest values within the test temperature range in terms of the same mixture gradation. For instance, at 55 °C, the dynamic moduli of asphalt mixtures with AC-20 gradation reached 97 MPa, 125 MPa, and 94 MPa for base asphalt mixtures, PB-modified mixtures and SBS-modified mixtures, respectively. These results indicate that the asphalt mixtures with modifier PB possess a superior deformation resistance at high temperature, which matches the results of both the wheel-tracking test and high-temperature creep test.

## 4. Statics Analysis of the Pavement Structure of Bus Lane

### 4.1. Pavement Structure Design of the Bus Lane

**Figure 12:**
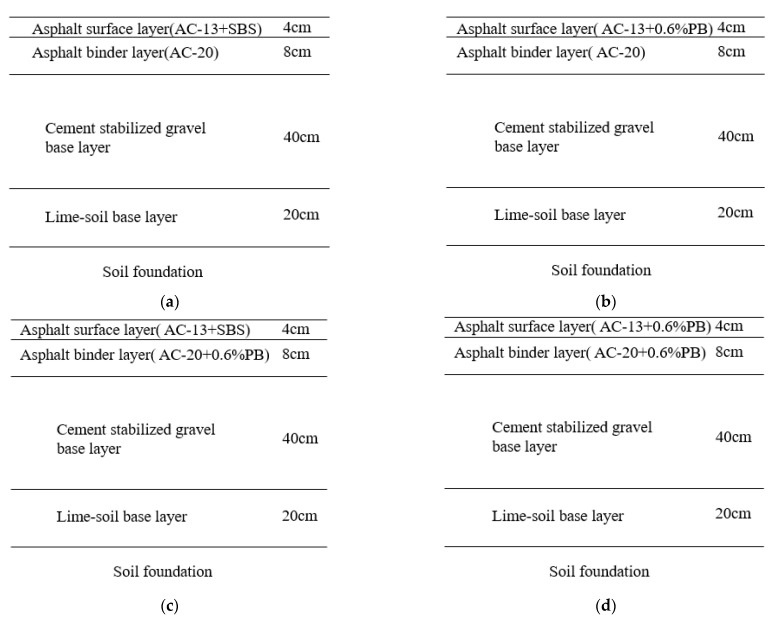
Pavement structure design of the bus lane. (**a**) Original pavement structure; (**b**) designed structure I; (**c**) designed structure II; (**d**) designed structure III.

Taking the pavement structure of the bus lane of Wenchang Road in Yangzhou City for example, the original pavement structure included five layers, namely (from top to bottom) an asphalt surface layer, asphalt binder layer, cement-stabilized gravel base layer, lime-soil base layer, and soil foundation. Asphalt mixtures with a gradation of AC-13 and AC-20 were used for the surface layer (4 cm thickness) and binder layer (8 cm thickness), respectively. PB-modified AC-13 and PB-modified AC-20 were used to replace the original materials of the asphalt surface or (and) binder layer. Therefore, three different types of pavement structures of the bus lane were proposed, as shown in Figure 12. The finite element software of ABAQUS 6.11 was employed to perform the statics analysis. The distribution of the corresponding stress and stain was analyzed for the designed structures of bus lane.

### 4.2. Material Parameters

The material parameters selected for the statics analysis are listed in Table 7, of which, the density and Poisson ratio were provided by Yangzhou City Municipal Administration Department. It should be noted that the dynamic modulus of the asphalt mixture at 0.1 Hz and 20 °C was used as the modulus needed in Table 7, though the static modulus of the asphalt mixture should have been used herein. This is because there is a good correlation between the dynamic modulus and static modulus of asphalt mixtures, and the values of the two indexes become comparable, especially at low-frequency conditions (such as 0.1 Hz) [37].

### 4.3. Load Determination

The standard tire grounding pressure of 0.7 MPa is commonly used for pavement structure design in China. However, the tire ground pressure of a typical city bus normally exceeds 0.7 MPa. Therefore, the actual load was utilized for this study, along with the standard axle load. According to the data provided by Yangzhou City Bus Company, as shown in Table 8, a tire ground pressure of 0.83 MPa was considered as the actual load of a typical type of bus in Yangzhou city.

Since a two-dimensional pavement model was used for the statics analysis, the applied load in the modeling process needs to be converted from the surface load (tire ground pressure) to line load. The relationship between the tire ground pressure and axle load can be expressed by Equation (9) [39,40].

(9) $$\frac{p_{i}}{p}={\left(\frac{L_{i}}{L}\right)}^{0.65}$$

Therefore, the axle weight of the vehicle can be calculated by Equation (10).

(10) $$L_{i}=L\sqrt[{13}]{{\left(\frac{p_{i}}{p}\right)}^{20}}$$

where: *p_i_* is the tire ground pressure, MPa; *p* is the standard tire ground pressure, 0.7 MPa; *L_i_* is the axle weight of the vehicle, kN; *L* is the standard axle load, 100 kN.

In addition, the equivalent circle radius of the tire contacting area can be calculated by Equation (11), based on which, the line load can be calculated by Equation (12).

(11) $$A=\frac{L_{i}/4}{p_{i}},\;r=\sqrt{\frac{A}{\pi }}$$

where: *A* is the tire contacting area, cm^2^; *r* is the equivalent circle radius, cm.

(12) $$q_{l}=\frac{L_{i}/4\times 10^{3}}{2r\times 10^{-2}}$$

where: *q_l_* is the line load, N/m. The results are listed in Table 9. 

### 4.4. Model Establishing

**Figure 13:**
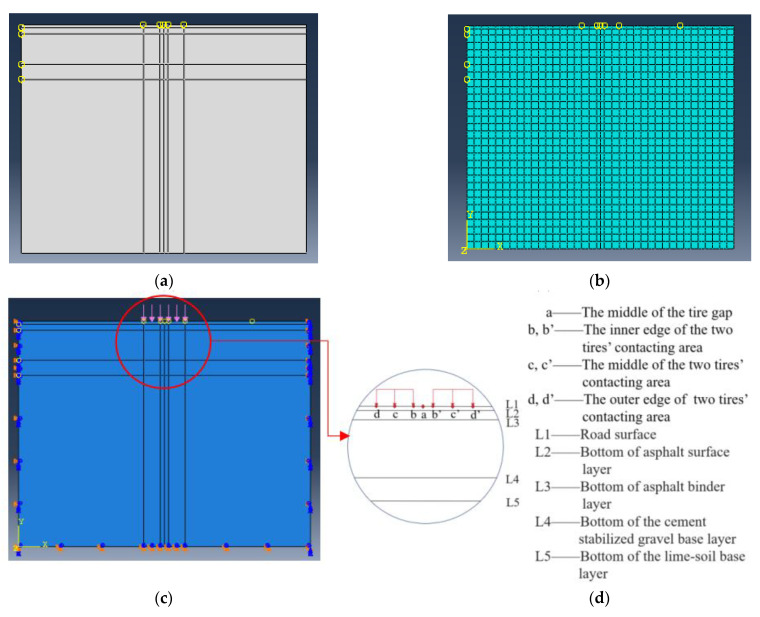
Model establishing process. (**a**) Modeling; (**b**) mesh division; (**c**) loading and boundary condition case; (**d**) pavement coordinate points and vertical positions.

A two-dimensional model of the cross-section of the bus lane was built for this study, with dimensions of 3.75 m (width) × 3 m (height). The CPE8R (reduced integral) unit was used. In order to accelerate the running process, the mesh was divided into a size of 0.1 m × 0.1 m for this calculation, along with meshes of 0.04 m × 0.1 m and 0.08 m × 0.1 m for the asphalt surface and binder layers, respectively. There are seven positions where the pavement is most likely to be damaged, which were marked as coordinate points in the model, namely the middle of the tire gap (Point a), the inner edge of the two tires’ contacting area (Point b and b’), the middle of the two tires’ contacting area (Point c and c’), and the outer edge of the two tires’ contacting area (Point d and d’). The model establishment, mesh division, and loading and boundary conditions are shown in Figure 13.

The following assumptions were taken for this calculation: (1) the materials of each structure layer are homogeneous and uniformly continuous; (2) the material parameters keep constant with the changing of time and temperature; (3) there is no transverse displacement on the left and right sides of the model, and no transverse and vertical displacement on the bottom surface as well.

### 4.5. Statics Simulation Results

#### 4.5.1. Tensile Stress Distribution

**Figure 14:**
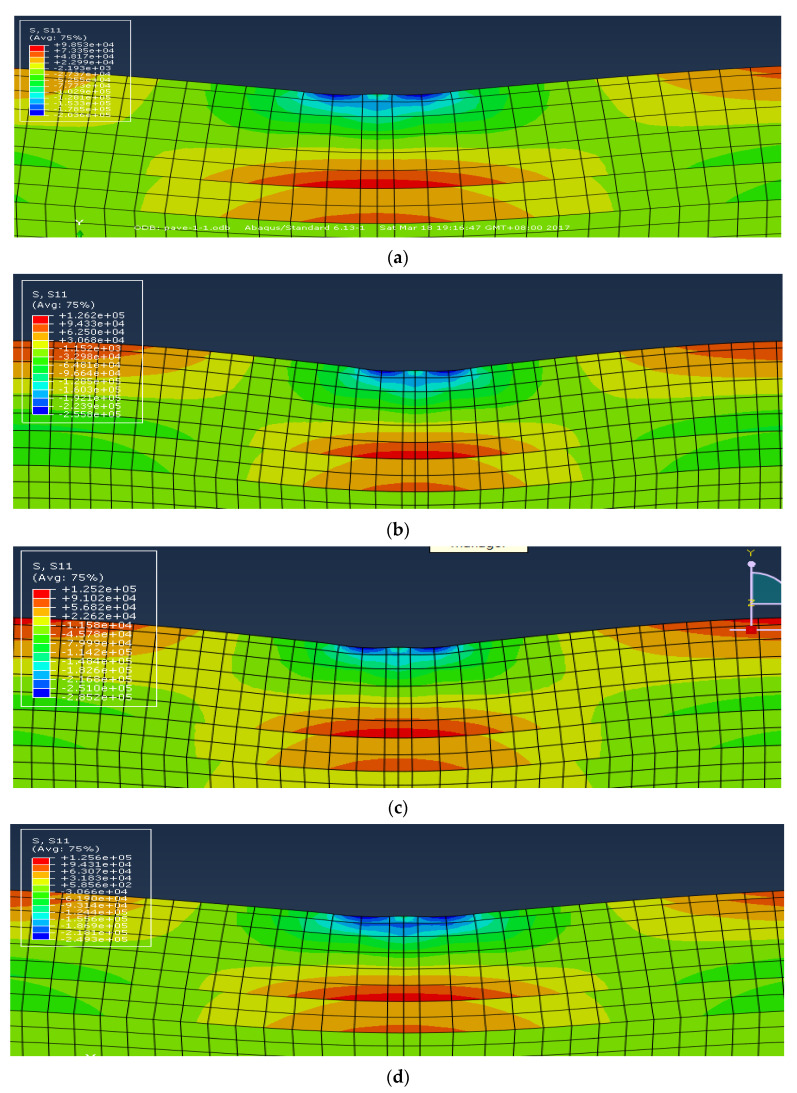

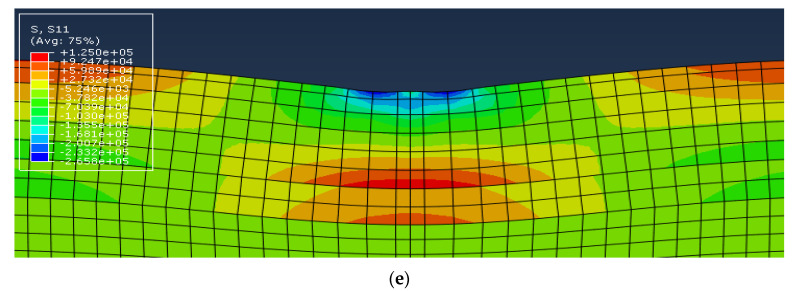
Tensile stress clouds of different pavement structures. (**a**) Tensile stress cloud of the original pavement structure under 0.7 MPa; (**b**) tensile stress cloud of the original pavement structure under 0.83 MPa; (**c**) tensile stress cloud of the designed structure I under 0.83 MPa; (**d**) tensile stress cloud of the designed structure II under 0.83 MPa; (**e**) tensile stress cloud of the designed structure III under 0.83 MPa.

**Figure 15:**
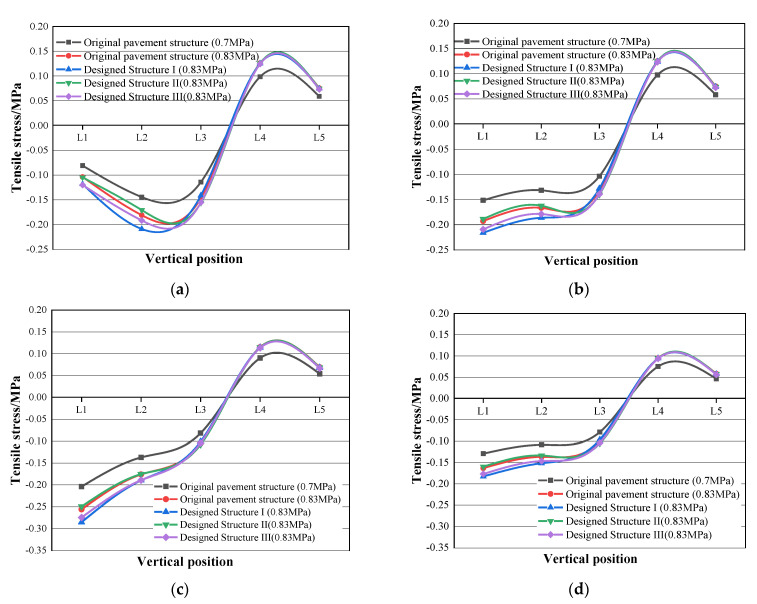
Maximum tensile stress of each potentially damaged point in different layers. (**a**) Maximum tensile stress at Point a; (**b**) maximum tensile stress at Point b; (**c**) maximum tensile stress at Point c; (**d**) maximum tensile stress at Point d.

Under both standard tire ground pressure (0.7 MPa) and actual tire ground pressure (0.83 MPa), the tensile stress clouds of the original pavement structure and the three types of designed structures are illustrated in Figure 14. The maximum tensile stress of each potentially damaged point (Point a to Point d), which appears at the bottom of each structure layer, is plotted with the different vertical positions (L1 to L5) in Figure 15. Due to the symmetry of the loading and the structure, only the data of one side (Point b, c and d) were used for analysis.

As shown in Figure 15, the tensile stress values of all coordinate points were negative on L1 to L3, but turned positive on L4 and L5. These results indicate that the road surface (L1) and the bottoms of the asphalt surface layer (L2) and binder layer (L3) endure compressive stresses, whereas the bottoms of the cement-stabilized gravel base layer (L4) and lime-soil base layer (L5) suffer tensile stresses. In addition, in the middle of the tire gap (Point a), the maximum compressive stress appeared on L2, as shown in Figure 15a. The maximum compressive stress at other coordinate points occurred on L1, as shown in Figure 15b–d.

It can also be observed that the axle load causes a significant impact on the tensile stress distributions. Taking the coordinate Point a of the original structure for example, as shown in Figure 15a, when the applied axle load increased from the standard tire ground pressure of 0.7 MPa to the actual tire ground pressure of 0.83 MPa, the tensile stresses on L1 to L5 increased by 28.3%, 25.3%, 27.9%, 28.0%, and 28.5%, respectively.

Furthermore, the use of PB-modified asphalt mixtures also resulted in a fluctuation in tensile stress distributions. As shown in Figure 15, compared with the original structure, the compressive stress on L1 to L3 increased to some extent when the PB-modified surface layer or (and) binder layer were designed, whereas the tensile stresses on L4 and L5 remained as the same value. This is due to the higher moduli of the asphalt mixtures with modifier PB, leading to the PB-modified layers bearing more stress. Since asphalt mixtures possess a superior compressive strength, the increasing compressive stress does not mean an accelerated deterioration of asphalt structure layers.

#### 4.5.2. Vertical Deformation

**Figure 16:**
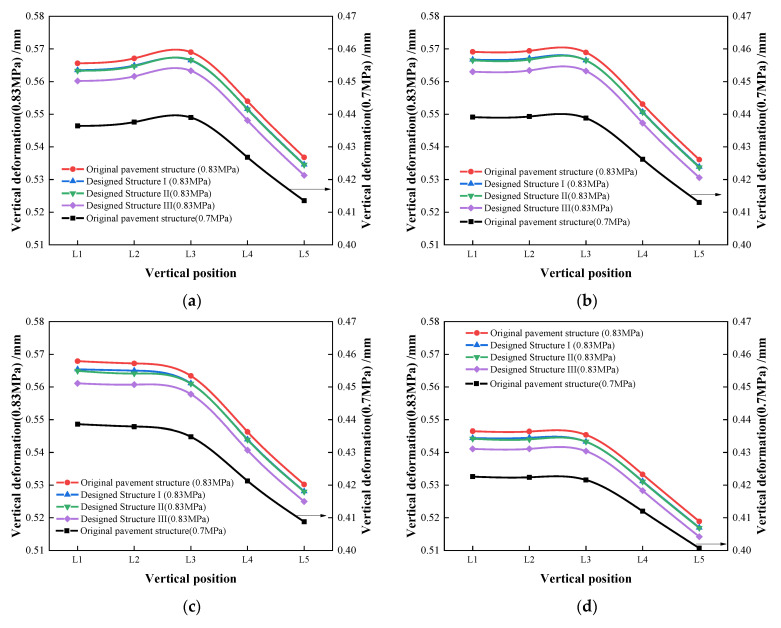
Vertical deformation of each potentially damaged point in different layers. (**a**) Vertical deformation at Point a; (**b**) vertical deformation at Point b; (**c**) vertical deformation at Point c; (**d**) vertical deformation at Point d.

Under tire ground pressures of both 0.7 MPa and 0.83 MPa, the vertical deformation of all of the potentially damaged points on L1 to L5 are illustrated in Figure 16. The vertical deformation of the asphalt pavement under load could reflect the deformation resistance of a pavement.

As shown in Figure 16, the tire ground pressure impacted the vertical deformation hugely. Taking the coordinate Point a of the original structure for example, when the tire ground pressure increased from 0.7 MPa up to 0.83 MPa, the vertical deformation increased by 29.5~29.8% on L1 to L5.

In addition, the use of the PB-modified surface layer or (and) binder layer could reduce the vertical deformations effectively compared with the original structure. It was also noticeable that designed structure III presented the smallest vertical deformation, followed by designed structure II and structure I. This result indicates that structure III possesses a superior resistance to deformation.

## 5. Conclusions

A new type of self-developed plastic and basalt fiber composite modifier called PB modifier was used to fabricate asphalt mixtures. A series of laboratory tests, including the wheel-tracking test, dynamic creep test, low-temperature bending beam test, immersion Marshall test, freeze–thaw splitting test, and dynamic modulus test, were adopted to explore the influence of the PB modifier on the performance of asphalt mixtures. In addition, three types of urban bus lane pavement structures were proposed by using the PB-modified asphalt mixtures for the surface layer and (or) binder layer. Statics analyses were conducted using ABAQUS 6.11 finite element software. According to the results and discussions in this study, the following conclusions can be drawn:

(1) Adding PB modifier can improve the dynamic modulus and high-temperature stability remarkably and reduce the creep rate of asphalt mixtures, presenting a superior high-temperature stability that is even better than SBS-modified asphalt mixtures.
(2) By combining with basalt fiber, the PB modifier can compensate for the adverse effect on the low-temperature crack resistance of mixtures caused by the addition of waste plastics.
(3) The PB modifier can not only improve the anti-water damage performance indexes of the residual stability and tensile strength ratio of mixtures, but can also strengthen the absolute values of the strengths, presenting a better water damage resistance.
(4) The actual axle load of a bus will cause severe tensile stress and vertical deformation compared with the standard axle load. Using PB-modified asphalt layers for bus lanes can offset the negative impact caused by a heavy axle load. Using PB-modified asphalt mixtures for both the surface layer and binder layer (designed structure III) presents the best strengthen function.

The research results of this paper provide a reference for the selection of the pavement materials and structures of the urban bus lane, which has certain theoretical significance and application value.

## Figures and Tables

**Table 1:** Compositions and the contents of waste plastic.

Resin	Polythene (PE)	Ethylene Vinyl Acetate Copolymer (EVA)	Coupling Agent	Auxiliary	Melting Point/°C
10~20%	65~75%	10%	2%	3%	90~100

**Table 2:** The properties of basalt fiber.

Index	Fracture Strength/MPa	Elongation at Break/%	Elastic Modulus/GPa
Value	2500–5000	2.69	90–110
Requirements in T/CHTS 10016	≥2000	≥2.1	≥80

**Table 3:** Technical indicators of the modifier PB.

Project	Appearance	Particle Size/mm	Length/mm	Density/g·cm^−3^
PB	External colorless transparent, internal brown, flat solid particles	2.5~3.5	6	1.82~1.86

**Table 4:** Technical properties of the mineral filler.

Index Items		Index Results	Index Requirements
Apparent density (g/cm^3^)		2.714	≥2.50
Water content (%)		0.38	≤1.0
Appearance		No clumps	No clumps
Water affinity coefficient		0.60	<1
Size range (%)	<0.6 mm	100	100
	<0.15 mm	100	90–100
	<0.075 mm	92.2	75–100

**Table 5:** Technical properties of the two types of asphalts.

Properties	Penetration (25 °C)/0.1 mm	Penetration Index PI	Softening Point /°C	Ductility (5 cm/min)/cm	Viscosity (135 °C)/Pa·s
Base asphalt	71.2	−0.8	47.1	150 (15 °C)	0.92
SBS-modified asphalt	67	0.3	78	48 (5 °C)	1.8

**Table 6:** Marshall test results of different asphalt mixtures.

Types of mixture	Optimum Asphalt Content (OAC)/%	Air Voids (VV)/%	Voids in Mineral Aggregate (VMA)/%	Voids Filled with Asphalt (VFA)/%	Marshall Stability/kN	Flow Value/0.1 mm
Base asphalt + AC-13	5.0	4.1	14.2	71.1	9.5	29.8
Base asphalt + AC-13 + PB	5.4	4.4	15.3	71.1	12.5	24.2
SBS-modified asphalt + AC-13	5.0	4.3	15.0	71.6	12.0	26.4
Base asphalt + AC-20	4.4	4.3	13.5	67.9	11.5	32.4
Base asphalt + AC-20 + PB	4.7	4.7	14.3	66.7	13.5	28.6
SBS-modified asphalt + AC-20	4.4	4.4	13.5	67.5	12.4	30.7

**Table 7:** Material parameters for statics analysis.

Structure Sheaf	Material	Density/kg·m^−3^	Modulus/MPa	Poisson Ratio
Surface layer	AC-13 + SBS	2360	1207	0.30
	AC-13 + 0.6% PB	2360	1438	0.30
Binder layer	AC-20	2450	1340	0.30
	AC-20 + SBS	2450	1260	0.30
	AC-20 + 0.6% PB	2450	1630	0.30
Base course	Cement-stabilized gravel	2200	1500	0.20
Sub-base	12% lime soil	2100	550	0.30
Soil base	soil	1800	45	0.40

**Table 8:** Technical parameters of a typical type of bus in Yangzhou city.

Index	Length/mm	Width/mm	Height/mm	Curb Weight/kg	Full Quality/kg	Tire Ground Pressure/MPa
Parameter	12,000	2550	3120	11,200	17,500	0.83

**Table 9:** Results of line load conversion.

Parameter	Tire Ground Pressure/MPa	Axle Load /kN	Equivalent Circle Radius/cm	Line Load /N·m^−1^
Standard axle load	0.7	100	10.65	117,371
Actual bus axle load	0.83	130	11.16	145,740

## Data Availability

Not applicable.
